# Surfacing undiagnosed disease: consideration, counting and coding

**DOI:** 10.3389/fped.2023.1283880

**Published:** 2023-10-25

**Authors:** Megan F. Baxter, Michele Hansen, Dylan Gration, Tudor Groza, Gareth Baynam

**Affiliations:** ^1^ Emergency Department, Perth Children’s Hospital, Perth, WA, Australia; ^2^ School of Medicine and Dentistry, Griffith University, Gold Coast, QLD, Australia; ^3^ Telethon Kids Institute, University of Western Australia, Perth, WA, Australia; ^4^ Western Australian Register of Developmental Anomalies, King Edward Memorial Hospital, Perth, WA, Australia; ^5^ Rare Care Centre, Perth Children’s Hospital, Perth, WA, Australia; ^6^ Undiagnosed Diseases Program, WA, Genetic Services of WA, Perth, WA, Australia

**Keywords:** rare disease, ICD-11, diagnostic odyssey, diagnostic coding, red flags, key timepoints

## Abstract

The diagnostic odyssey for people living with rare diseases (PLWRD) is often prolonged for myriad reasons including an initial failure to consider rare disease and challenges to systemically and systematically identifying and tracking undiagnosed diseases across the diagnostic journey. This often results in isolation, uncertainty, a delay to targeted treatments and increase in risk of complications with significant consequences for patient and family wellbeing. This article aims to highlight key time points to consider a rare disease diagnosis along with elements to consider in the potential operational classification for undiagnosed rare diseases during the diagnostic odyssey. We discuss the need to create a coding framework that traverses all stages of the diagnostic odyssey for PLWRD along with the potential benefits this will have to PLWRD and the wider community.

## 1. Introduction

Rare diseases (RD) are conditions with a specific pattern of clinical signs, symptoms, and findings that affect fewer than 1 in 2,000 persons living in any World Health Organisation-defined region of the world (1). There are more than 7,000 rare diseases, largely genetic (>80%) in origin, with more than half affecting children (2, 3). Whilst rare diseases individually represent a small population burden, collectively they represent a much larger healthcare burden with between 3.5% and 5.9% of the world's population living with a rare disease, equating to an estimated 262.9–446.2 million people globally (3). The direct and indirect costs of only a subset of rare diseases in the USA alone is approximately $1 trillion p.a. and two thirds of national paediatric inpatient expenditure (105 Billion p.a. RD vs. 70 billion p.a. for common diseases) (4, 5). Early and accurate rare disease diagnosis is critical to ensure that there is informed decision making, targeted treatments, reduced complication risks and improved patient wellbeing (6) and healthcare sustainability. However, people living with a rare disease often share a long arduous diagnostic process with a mean time to a diagnosis between 5 and 7 years (7, 8). Every rare disease starts out undiagnosed, and currently globally the majority of people living with a rare disease (PLWRD) are undiagnosed. Many, likely the majority, are not referred for a specialist assessment nor get access to (genomic) diagnostics. Even for those referred and assessed in specialist centres, the typical diagnostic rate is approximately 50% (9). The individual burden and population magnitude of impact of undiagnosed rare disease necessitates measures to improve rare diseases diagnosis, however the fundamental issue of defining a systematic approach to surfacing potential undiagnosed rare diseases and the related coding approach for undiagnosed rare diseases that can be applied to different data sets at various points in the diagnostic odyssey remains to be comprehensively addressed.

If you don't look for something, you will not find it. Colloquially, rare diseases are often referred to as “zebras” with advice given to clinicians to not consider rare diseases first; “When you hear hoofbeats behind you, don’t expect to see a zebra” (10). Focusing on individually common diseases first, or exclusively, prolongs the diagnostic process. Similarly, there is a systemic bias that hinders tracking the undiagnosed due to the dearth of specific and systematically unified processes and codes for a suspected or likely undiagnosed rare disease. If you can't measure it, you can't improve it. ICD-10 limitations for rare diseases coding are well documented (11, 12). When developing ICD-11 the WHO established Topic Advisory Groups, including a Rare Disease Group, ensuring the ICD-11 coding system had a greater focus on rare disease. By utilising the Orphanet database, and a poly-hierarchal classification structure the ICD-11 includes a unique identifier for classification of over 5,500 rare diseases (11) It also has a dedicated code MG48—“unknown and unspecified causes of morbidity”, similar to the Orphanet code ORPHA: 616874—“rare disease without a determined diagnosis after full investigation”. The IDC-11 code is only applicable after extensive specialist assessments are completed and neither codes alone can be used to track across the journey prior to such an assessment (11, 13). This means that often for numerous years, pending specialist assessments, if these in fact occur, undiagnosed individuals are invisible within systems with no comprehensive or consistent identification, flags for consideration, or operational definitions of “suspected” or “likely” undiagnosed rare disease. Accordingly, there is an inability to identify and track the hundreds of millions of people living with undiagnosed rare diseases. This prohibits health, and other system planning for this enormous group of people, which in turn threatens the value and sustainability of healthcare. Herein, we apply an Australian lens whilst noting that much of the below is generalizable internationally.

Herein we:

1. Highlight key time points and flags for considering a rare disease diagnosis, and
2. Provide thematic suggestions of some elements or characteristics that could help inform the definition of codes along the diagnostic process.

We do not endeavour to create a definitive list of codes or their elements, but seek to both raise awareness of the need and deliver an initial framework for considering an approach to surfacing and monitoring undiagnosed rare diseases.

## 2. Benefits of shortened diagnostic odyssey

There are numerous benefits to shortening the diagnostic odyssey. From a patient perspective there are psychological, social and functional implications (14). Earlier diagnosis allows for better informed decision making with the patients able to access more accurate information with regards to likely progression and potential disease symptoms along with more accurate life expectancy information. Given these diseases are largely genetic, patients and their families are also able to access better information to support further family planning. This includes correct information from a genetic counselling perspective with regards to the risk of existing siblings and further children being affected and the option to access carrier screening or preimplantation genetic testing if desired (7). Patients and families are also better able to access appropriate targeted support and advocacy groups (15). Overall, shortening the diagnostic odyssey can significantly improve patients quality of life (14).

With regards to investigations, a specific diagnosis allows for the minimization of unnecessary, often painful, tests and assessments with the focus shifting to monitoring disease (16).

From a treatment perspective a single diagnosis allows patients to make more informed decisions with regards to treatment. Earlier disease identification can result in an increased range of treatments available. It also ensures that specific targeted treatments can be offered when available rather than just symptomatic management and can prevent unnecessary, ineffective treatments from being commenced. Finally, it allows for consideration of inclusion in appropriate clinical trials and research groups for which patients are often unlikely to meet inclusion criteria without a formal diagnosis (17).

All of these benefits of earlier disease diagnosis also result in a reduced healthcare burden allowing for streamlining of healthcare services, having a significant fiscal benefit (18, 19).

## 3. Elements that could flag the consideration of a rare disease

The following elements can be considered in the diagnosis of rare diseases (see Table 1):

**Table 1:** A framework of potential flags for consideration of an undiagnosed rare disease over time.

Timepoint	Indicators	Data source
Prenatal	Abnormal prenatal screening tests	Laboratory Testing
	Multiple Congenital Anomalies	Clinical Records
	Congenital Anomaly and teratogen exposure	Clinical Records & Patient History
	Congenital Anomaly and history of difficulty conceiving and/or recurrent miscarriage	Clinical Records & Patient History
Newborn	Family GENES	Clinical Records
	Abnormal newborn screening result e.g., neonatal blood spot screen, congenital hearing loss, congenital cardiac disease	Laboratory Testing
	NICU Admission, especially combined with additional factors e.g., Family GENES	Hospital administrative data & Clinical records
	Prolonged NICU admission	Hospital administrative data
Early childhood	High number of primary care appointments	Primary care records
	Failure to reach milestones and/or regression in milestones	Clinical records
	Length of hospital stay	Hospital administrative data
	High number of ED presentations	Hospital administrative data
	Multiple specialist appointments, especially with multiple specialty types	Hospital administrative data
	School concerns	Patient history & school reports
Puberty and transition to adult care	Precocious puberty, delayed puberty, phenotype exacerbation	Clinical Records & Patient History
Childhood death and palliative care	Palliative care referral	Clinical Records & Hospital Administrative data
	Childhood death (individual or recurrent within a family)	Clinical Records

NICU, neonatal intensive care unit; ED, emergency department.

### 3.1. Clinical phenotypic factors

The mnemonic “Family GENES” has been proposed in order to identify risk factors for rare disease (20); Family—history with multiple generations or siblings affected with a similar phenotype

G—Groups of congenital abnormalities
E—Extremes or exceptional presentations of common disease
N—Neurodevelopmental delay or regression
E—Extremes or exceptional pathology
S—Surprising lab values

This mnemonic broadly highlights clinical red flags to consider, but does not reflect key timepoints at which to consider them, with one of the main barriers to rare disease diagnosis being the initial consideration of the possibility of a rare disease. This approach can be applied “manually” through care pathways or converted to algorithmic approaches that can be applied automatically from electronic health records. However, it may not be readily applicable to existing e.g., administrative data sets which may lack these data elements.

### 3.2. Administrative data elements

The often-complex nature and high burden of rare diseases generates flags that could be identified in administrative data, these include:

• Presentation to multiple specialty types/multisystem disorder.
• Multiple investigations, and multiple types of investigations.
• Frequent inpatient and outpatient admissions.
• Multiple medications, especially in children.
• Frequent emergency department presentations.
• High total medical treatment cost per child.
• Multiple surgeries.
• Death, especially in children.
• Frequent mental health presentations.

## 4. Timepoints at which to consider a rare disease diagnosis and related flags

### 4.1. Antenatal

Abnormal prenatal screening tests, such as biochemistry, imaging and molecular tests may prompt the consideration of diagnostic testing to detect a rare, and often genetic, disease cause. Individual morphological factors, such as intrauterine growth restriction (IUGR), may be indicators. Between 5% and 20% of IUGR is the result of an underlying genetic cause, in particular symmetrical IUGR, the result of early pregnancy growth restriction (21). Further, there may be a greater index of suspicion for a rare disease in the presence of multiple congenital anomalies, or with a history of difficulty conceiving or of recurrent miscarriage and a structural anomaly suggesting the possibility of a familial chromosomal rearrangement (22). Additionally, a history of teratogen exposure e.g., valproate should prompt the consideration of a rare disease (23).

### 4.2. Puerperal and Neonatal

The average hospital admission at time of birth is 6–48 h following a vaginal delivery and 3 days following a caesarean section (21). This time-period represents one of the key touchpoints where extensive assessments can be completed.

#### 4.2.1. Dysmorphology assessment

In infants with congenital anomalies +/− facial dysmorphism clinical gestalt can lead to a key differential diagnosis and further clinical assessment and testing (24).

#### 4.2.2. Routine screening

The Newborn Bloodspot Screening (NBS) test is completed between 48 and 72 h after birth (25).The test forms part of a screening process assessing for severe rare disease for which early identification significantly improves management. Formal confirmatory diagnostic testing should be considered for any positive results.

Newborn hearing screening (NHS) in Australia is performed by otoacoustic emission testing prior to hospital discharge (26). Eighty percent of prelingual deafness is genetic and rare and there are over 400 genetic syndromes that include hearing loss (27, 28). If an infant fails the otoacoustic emission testing, it is worth not only performing further audiology testing but considering other underlying conditions. Failure of NHS raises a high index of suspicion for a rare disease, especially if there are other red flags.

#### 4.2.3. NICU admission

In Australia, 18% of infants require an admission to a Neonatal Intensive Care Unit (NICU) or Special Care Nursery (SCN) (21). Of those patients admitted to the NICU up to 13% have been estimated to have a congenital malformation which may indicate an underlying rare disease (28).

#### 4.2.4. Symptoms

The following symptoms in a newborn should all be assessed to identify if further investigation is required: difficulty feeding, respiratory distress, and abnormal muscle tone. For example, difficulty feeding is often multifactorial in nature but can be due to craniofacial abnormalities, brainstem dysfunction and poor coordination along with aspiration and malabsorption (29).

### 4.3. Early childhood

#### 4.3.1. Primary care

A Primary Care Provider (PCP) creates a common touch point for healthcare of PLWRD. PCPs are often the first to identify a potential underlying problem and document any changes over time. On average all children visit a General Practitioner (GP) 3.8 times per year (30). With 80% of children diagnosed with a rare disease visiting their GP at least once in the previous 12-month period (31). It is important to consider any underlying conditions during routine appointments including vaccinations and baby checks, and frequent GP visits alone may be an indicator of rare disease. It is also worth considering a rare disease in the setting of making multiple different specialist referrals in a short time-period.

A rare disease cause should be considered when there is a delay in reaching milestones in one or more domains, or loss or regression of skills. For example, when metabolic pathways are affected resulting in toxic metabolites as in Tay-Sachs Disease, or abnormal protein production preventing development as in Rett Syndrome, there is regression of skills (32). It is also important to consider in cases of extremes of growth or change in growth centiles, for example Noonan or Silver-Russell syndrome in short stature or Sotos syndrome with a larger stature (33–35). The Family GENES mnemonic provides a useful framework for Primary Care (20).

#### 4.3.2. Hospital

Despite having significantly less granularity than clinical records, administrative data can often provide insights for further investigations. When admitted to hospital, patients with rare diseases have a significantly increased duration of hospitalisation, increased cost of care and larger number of procedures. Hence rare diseases should be considered in anyone who has a length of stay greater than one standard deviation from the mean within a given department (4, 36). This disparity is seen to a lesser extent in emergency departments however children with rare diseases are still over represented (4). Any child with 3 or more ED presentations in a one-year period requires consideration for an underlying disorder.

**Figure 1:**
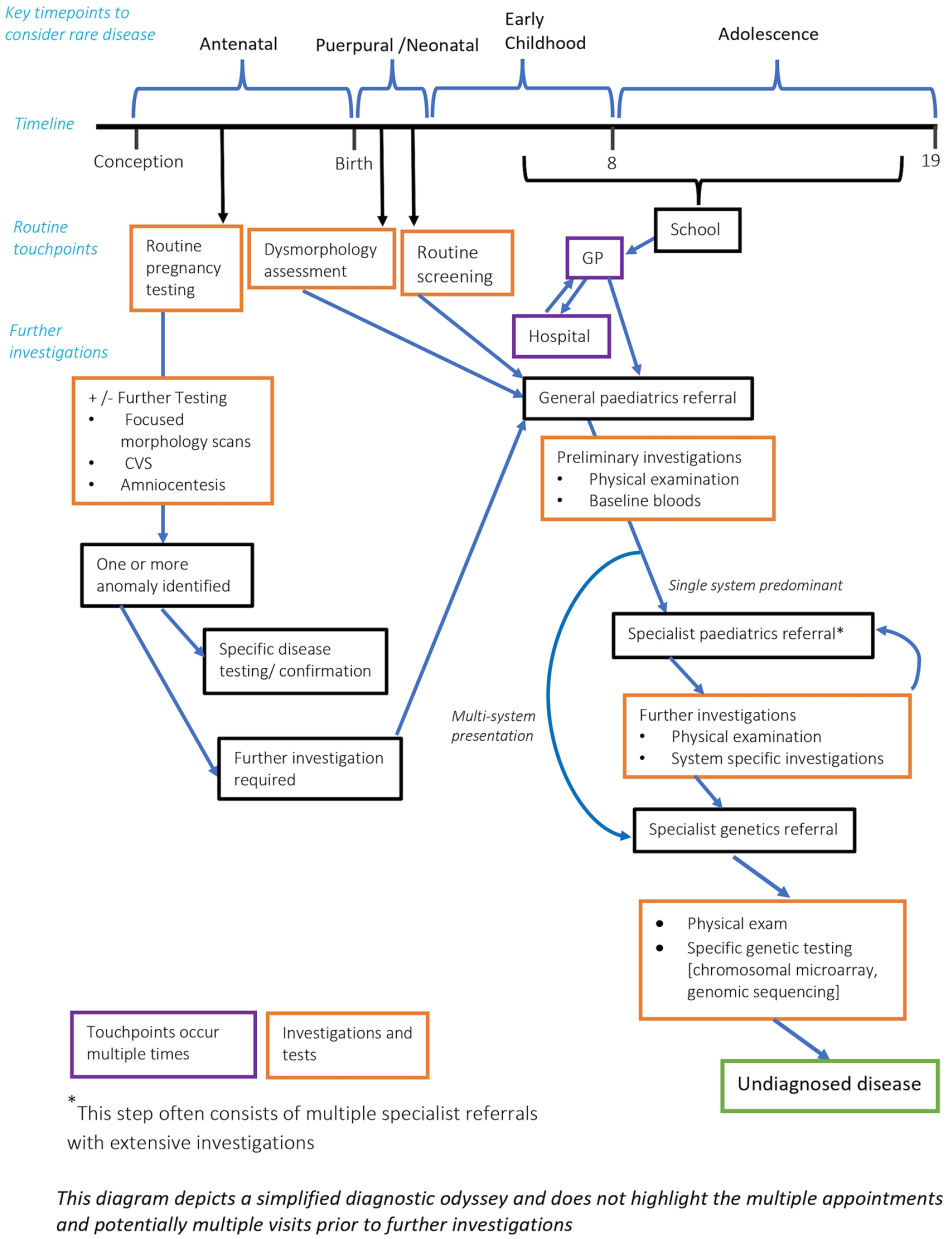
Timepoints to consider a rare disease diagnosis.

Outside of patients with rare disease, the average number of specialists seen is very low. In patients with a possible rare disease, the number of specialists can be significantly higher given the multiorgan involvement and prolonged diagnostic period. In a cohort study for patients diagnosed with a mitochondrial disease the average number of doctors seen was 8.19 (37). Hence in patients that have 2 or more active specialists with distinct organ pathology, or 3 or more distinct specialists in early childhood, rare disease should be considered. It is worth noting that multiple specialist referrals and investigations is often a significant rate limiting component to the diagnostic process (see Figure 1).

#### 4.3.3. School

Given the extensive amount of time a child spends in the classroom, teacher's concerns should be noted. Failure to achieve milestones in developmental domains, consistently poor assessments, significantly small stature, hearing difficulties or vision issues, functional limitations, or strikingly abnormal behaviours should all prompt the need for further assessment.

### 4.4. Adolescence and transition to adult care

Whilst many rare diseases present in early childhood, they may also affect puberty. For example, patients with neurofibromatosis Type 1 have a significantly higher rate of precocious puberty than the general population. In cases of both peripheral and central precocious puberty an underlying genetic syndrome should be considered (38). This is also true in cases of delayed puberty and primary amenorrhoea (39). An individual's phenotype can also be exacerbated during puberty.

Transition to adult care provides another opportunity to revisit diagnosis. Children with complex undiagnosed diseases undergoing transition to adult services may not have had a diagnostic assessment for several years, and in that interval new diseases will have been identified and diagnostic techniques will have advanced (40). Similarly, those with a clinically diagnosed disorder, such as cerebral palsy, may not have had an assessment for an underlying rare genetic disease cause. Many children in transition clinics are likely to have an (undiagnosed) rare disease.

### 4.5. Childhood death and palliative care

Six in 10 deaths in children are due to rare diseases (41); rare diseases are the biggest killer of children in high income countries. Childhood death and palliative care are red flags for rare diseases. Any child without a confirmed diagnosis of a rare disease entering palliative care should be considered for diagnostic review, as should any child with a phenotype, such as cerebral palsy, which has a significant likelihood of an underlying rare disease. The possibility of a rare disease should be considered for any childhood death, especially unexplained and/or associated with other red flags for rare diseases.

A specific focus on considering rare disease at key timepoints is one of the ways the diagnostic odyssey will be better addressed.

## 5. The need for unified explanatory diagnosis classification during the diagnostic process

Currently there are significant multi-stakeholder challenges arising from the lack of systematic and comprehensive classification and coding along the patient journey:

### 5.1. Patients and their families

The diagnostic odyssey is often very discouraging to patients and their families, having a significant psychological burden (42, 43), and recurrently having to re-tell their history, especially in the absence of a diagnosis or descriptor that supports some level of validation and the opportunity for access to a tailored care pathway. A lack of provisional descriptor or a standardised code can also result in difficulties accessing the appropriate support groups and may impact access to financial support such as further access to disability services. For example, in Australia, National Disability Insurance Scheme (NDIS) often requires a diagnosis for access.

### 5.2. Healthcare workers

A single descriptive code that is not tailored to the nuances of rare disease is often selected for a patient's presentation. Given that many rare conditions are associated with numerous different symptoms, this can result in incorrect or incomplete coding with downstream consequences for inappropriate and fragmented treatment by the primary managing clinician. This also has implications for other healthcare workers as it does not convey that a currently unspecified disease is being considered, or should be considered.

### 5.3. Research

It is very difficult to identify all of the relevant patient data for rare diseases research because many of the presentations have been classified incorrectly in administrative data. From an epidemiological perspective, it is difficult to identify the burden of rare diseases; and from an individual disease perspective, it is difficult to obtain a complete cohort (44).

### 5.4. Hospital and government

As a result of difficulty assessing the burden of rare disease, funding cannot be allocated appropriately to patients or hospitals. This has significant implications with regards to staffing allocation, funding for investigations and health system planning and sustainability (44).

## 6. Functional classification at different stages of the diagnostic odyssey

As outlined above, there would be significant benefits to a universal classification approach that can be operationalized at various stages in the diagnostic odyssey for those with undiagnosed diseases. Currently within the hospital setting the coding process is laborious with clinical coders assigning codes from clinical notes including discharge summaries, results and patient notes. If specific criteria are met, as outlined in ICD 10 and now ICD ll, then a clinical code is assigned. This coding information is used to access activity-based funding and reimbursement and as such is a primary focus, however clinical coding is imperative to economic modeling, healthcare planning, along with research and education. It is important to note that if the specific criteria are not met or there is any uncertainty in the diagnosis then the coding is not applied. For example, it is possible to code notes citing “presumed”, “probable” or “definitive disease”. It is not possible to code “differential diagnosis”, “?” or “suspected” from within clinical notes (45). If the information is not present in the appropriate format within clinicians’ notes then no coding record of that part of the presentation or disease can be completed by clinical coders. Hence, the quality of documented information affects the quality of coded data (46).

From a rare disease perspective for a universal classification to be used during the diagnostic process it will require time to be developed and subsequently implemented in health systems. Whilst this is occurring, one interim approach is to adapt the use of existing e.g., ICD-10, or incoming e.g., ICD-11 coding, approaches combined with elements from the Orphanet nomenclature.

Both ICD-10 (R69) and ICD-11 (MG48) include a unique code to identify “unknown and unspecified causes of mortality and morbidity for use in undiagnosed diseases, not specified at the site or system involved”. A challenge to implementing and making inferences from these codes in isolation, is that they provide no information as to the nature or extent of diagnostic assessments that have been performed or the severity of the disease and affected organs. They do not allow for identification of a working diagnosis and the degree of diagnostic certainty associated with that.

The ICD-11 classification improves on ICD-10 by using multiple stem codes, or stem and extension codes, to define a patient's presentation. The stem codes provide information as to the clinical entity and can exist alone as a diagnosis, with the extension codes providing important additional descriptive information about a diagnosis (47). It is possible to use a stem on its own, multiple stems joined using a forward slash “/”, a stem and an extension joined using ampersand “&”, a stem and multiple extensions or multiple stems and multiple extensions joined using “/ and &”. For example, if a patient presented with moderate severity diabetic ketoacidosis with T1DM the following code could be used 5A22.0&XS0T/5A10, in which 5A22.0 is diabetic ketoacidosis without coma and XS0T is moderate severity and 5A10 is T1DM. Extension codes can provide information about anatomy and topography, laterality, aetiology, severity (e.g., stages of cancer diagnoses or mild, moderate, profound hearing loss), temporality, diagnosis method of confirmation (laboratory, genetics, imaging, etc.) and to a limited extent diagnostic certainty.

ICD-11 incorporates specific codes for all the rare diseases currently included in the Orphanet nomenclature. For example, the code LCD2F.15 represents Noonan syndrome for which there is no specific code in ICD-10. For this ICD-11 code to be utilised a formal diagnosis must be reached. However, this code is under the parent code LCD2F; syndromes with multiple structural abnormalities, without predominant body system involvement—which could be used, either alone or in combination with other data, as part of a coding system to help flag a possible undiagnosed rare disease.

There are some limitations of the ICD-11 classification for coding undiagnosed diseases. For example, it does not allow for a diagnostic assertion beyond the extension code “diagnostic certainty” which provides only two options—“provisional diagnosis” (XY7Z) and “differential diagnosis” (XY75). The Orphanet Nomenclature advocates using a diagnostic assertion whenever possible in coding cases of rare (or suspected rare) disease. This means a case with suspected Noonan syndrome awaiting further confirmation would be coded using the Orpha code for Noonan Syndrome with the diagnostic assertion “Suspected rare disease” equivalent to the ICD11 extension code “provisional diagnosis”. If the diagnosis is confirmed (i.e., there is sufficient diagnostic and/or clinical evidence), the case would be coded to Noonan Syndrome with the diagnostic assertion “Confirmed rare disease”. Where a physician is unable to determine a clinical diagnosis because of the absence of suitable tests or non-contributory tests the diagnostic assertion is “Undetermined (unknown) diagnosis”. The latter has been assigned a specific code in the Orphanet Rare Disease Classification System—ORPHA: 616874. This code is not meant for use in coding patients along their diagnostic pathway and should only be used after all reasonable efforts to obtain a diagnosis according to the best diagnostic capabilities available have been performed. The addition of diagnostic assertion coding should help differentiate cases of likely undiagnosed rare disease requiring access or referral to diagnostic services from likely undiagnosed rare disease despite extensive specialist assessment.

With an updated more extensive clinical coding system it remains more imperative than ever that clear notes outlining clinicians' thinking including likely diagnosis along with diagnostic certainty and information regarding extension codes are completed to allow for improved clinical coding.

## 7. Conclusion

Reducing the diagnostic odyssey for rare diseases is critical to improving care for PLWRD and their families. This can be enabled through early consideration of rare disease along with an improved coding framework during the diagnostic process. Looking towards the future, existing coding systems could be adapted, whilst supporting and awaiting the development of new coding methods that ultimately will facilitate linking those with undiagnosed diseases to better care pathways and outcomes.
